# Parental Experiences of Supporting Children with Clinically Significant Post-Traumatic Distress: a Qualitative Study of Families Accessing Psychological Services

**DOI:** 10.1007/s40653-017-0158-8

**Published:** 2017-06-19

**Authors:** Victoria Williamson, Cathy Creswell, Ian Butler, Hope Christie, Sarah L. Halligan

**Affiliations:** 1 Department of Psychology, University of Bath, Bath, BA2 7AY UK; 2 School of Psychology and Clinical Language Sciences, University of Reading, Reading, RG6 6UA UK; 3 Department of Humanities and Social Sciences, University of Bath, Bath, BA2 7AY UK; 4 Department of Psychiatry and Mental Health, University of Cape Town, Cape Town, 7700 South Africa

**Keywords:** Post-traumatic stress disorder, Trauma, Childhood, Parenting, Qualitative

## Abstract

The aim of this study was to investigate the experiences of parents in providing support to their child following trauma exposure in cases where children are experiencing clinically significant levels of post-traumatic distress. Qualitative interviews were conducted with parents whose child was exposed to a trauma and referred for psychological treatment. Parents reported considerable anxiety in coping with their child’s post-traumatic distress. Avoidance of trauma-related discussions was encouraged due to concerns that non-avoidant approaches may worsen children’s post-trauma difficulties. Nonetheless, parents were often sensitive to their child’s distress and offered reassurance and other forms of support. Many barriers existed to accessing psychological treatment, and perceptions of inadequate guidance from therapists on supporting child adjustment contributed to parental distress. The results illustrate the strategies used by parents in supporting their child post-trauma and may assist mental health professionals in providing acceptable guidance to parents following child trauma.

A significant number of children develop psychological adjustment difficulties such as post-traumatic stress symptoms (PTSS) following exposure to trauma (De Vries et al. 1999; Meiser-Stedman et al. 2006; Stallard et al. 2004). Parents are often children’s main source of support following a trauma and it is thought that parents’ responses can reduce or exacerbate their child’s vulnerability to PTSS (Scheering and Zeanah 2001). Several post-trauma parenting behaviours have been found to be significantly associated with the onset of childhood post-traumatic stress disorder (PTSD), including a lack of parental support (Bokszczanin 2008; Vernberg et al. 1996) and parental overprotection (Henry et al. 2004; Williamson et al. 2017). Conversely, providing children with opportunities to talk about the trauma and feel understood may be beneficial (Stallard et al. 2004).

Existing research has provided in depth exploration of parental experiences of providing support to children after single-incident trauma (Alisic et al. 2012; Williamson et al. 2016). These qualitative studies have highlighted that parents are sensitive to their child’s post-trauma distress and offer children reassurance and opportunities to discuss the trauma. Furthermore, efforts were made to resume children’s pre-trauma routines as a strategy to support child adjustment; however, parental anxiety that their child may re-experience illness or injury could inhibit the reinstatement of such routines.

However, little is known about the experiences and challenges faced by parents in supporting a child who is experiencing clinically significant symptoms and post-traumatic distress following trauma exposure. Supporting a child who is experiencing serious or persistent post-traumatic distress may be uniquely challenging. For example, worries about causing actual harm to the child by reminding them of the event are expressed by some parents in existing qualitative studies (Williamson et al. 2016), but may be much more significant where children are visibly struggling to cope. Moreover, having a child experience a life-threatening event can have direct psychological consequences for the parent, even when they themselves were not directly exposed to the trauma, and parental posttraumatic distress is greater in cases where the child is more seriously impacted (Hiller et al. 2016). Parental distress and PTSD symptoms have been found to be associated with poorer child adjustment following trauma exposure (Alisic et al. 2011; De Vries et al. 1999; Kelley et al. 2010; Nugent et al. 2007). Parental post-trauma distress stemming from child trauma exposure may cause parents to be less available to their child, lead to the promotion of maladaptive coping strategies, or cause parental difficulties in discussing the event which may obstruct child recovery (Nugent et al. 2007; Schwartz et al. 1994)

The limited available evidence suggests that only a small minority of children with PTSD access treatment (De Vries et al. 1999), and parents are likely to be an important determinant of this. Moreover, parental involvement in child psychotherapy may not only influence drop-out rates but parents may also foster child adjustment by helping children to practice therapeutic coping strategies at home (Chowdhury and Pancha 2011; Cobham et al. 2016; Schneider et al. 2013). However, parental perceptions of and engagement with their child’s psychological treatment post-trauma have received limited research attention (e.g. Salloum et al. 2014). A better understanding of parents’ views of providing support to a child with psychological adjustment difficulties following a traumatic event and views about psychological services may enable mental health professionals to provide advice and support that is acceptable and meaningful to parents following child trauma.

In order to gain a better understanding of parental experiences of child trauma and posttraumatic distress, we used in depth, qualitative methods to explore: parents’ experiences of caring for a child who is experiencing clinically significant distress following trauma exposure; the impact of child trauma and PTSS on the family more broadly; and experiences of accessing and engaging with psychological treatment for their child post-trauma.

## Method

The study received approval from the National Health Service Research Ethics Committee, Reading University Ethics Committee, and University of Bath Ethics Committee. All participants provided informed consent (parent) or assent (child) prior to participation.

### Participants

Six parents and seven children who experienced a traumatic event were recruited following the child’s attendance at Child and Adolescent Mental Health Services (CAMHS) in two National Health Service Trusts in England, or at the Child Bereavement, Trauma, and Emotional Wellbeing Service (CHUMS), a charitable organization providing psychological treatment to children following trauma exposure. In one case, two children in a single family had been exposed to the traumatic event and both children and their parent participated in this study. The age range of participating children was 6–16 years. The clinical care team contacted potentially eligible parents of children who received treatment from CAMHS or CHUMS following a traumatic event, and parents were contacted by researchers with further information following parental permission. The following exclusion criteria were used: existing organic brain damage or intellectual disability in the child that precludes mainstream schooling; parent or child inability to speak English; child registered with child protection services; and concerns that the respondent parent was the perpetrator of the trauma. Families were given a £10 voucher following their participation in the study. Of the 16 families approached, 10 did not participate in the study. Parents who did not participate either became uncontactable or reported that family members were uncomfortable with their participation.

### Assessments

Parents were invited to complete an in-depth, qualitative interview via telephone as the main outcome measure. In addition, in order to describe the sample, participating parents and their children completed questionnaire assessments of their psychological adjustment, either by post or online. In two cases the child did not complete the self-report questionnaires due to parental refusal.

#### Qualitative Data Generation and Analysis

The interview topic guide was developed in line with the research questions and literature regarding child and parent experiences and behaviours post-trauma. Following the collection of background information, parents were prompted to respond to interview topics related to their thoughts, feelings, and behaviours following the trauma; concerns about their child; their experiences of providing support for their child post-trauma; and perceptions of their child’s psychological treatment. Suggestions for improvements to the support available to parents, children, or families post-trauma were also discussed.

Respondent validation was conducted to increase the reliability and accuracy of the data through participant feedback (Lincoln and Guba 1985; Torrance 2012). Parents were provided with a summary of the key interview findings and preliminary interpretations following the interview. All input from participants regarding the interview summary was treated as additional data.

All interviews were transcribed verbatim and transcripts were entered into NVivo 10 (www.qsrinternational.com/products_nvivo.aspx) to facilitate analysis. Qualitative analysis of the interview transcripts was carried out using thematic analysis (Braun and Clarke 2006). Transcripts were read multiple times to facilitate familiarity with the data and coding was conducted systematically across the data set by the primary researcher. Potential themes were then abstracted from the codes (Patton 1980) with themes being representative of repeated patterns of meaning across the data set (Braun and Clarke 2006). Themes were reviewed for coherence through an examination of all coded text segments for each candidate theme, and consideration of whether themes accurately and distinctly reflect the meanings evident in the data (Attride-Stirling 2001; Braun and Clarke 2006). To ensure the credibility of the analysis, reflective memos were written during data analysis by the primary researcher to keep a record early interpretations of the data and relationships between concepts (Birks et al. 2008; Whittemore et al. 2001). A reflexive journal was kept throughout data collection and analysis in an effort to recognize the influence of the researcher’s prior experiences, thoughts, and assumptions and prevent premature or biased interpretations of the data (Mason 2002; Morrow 2005).

As it is possible for different interpretations to be made during data analysis depending on the beliefs and knowledge background of the researcher, to ensure criticality and integrity, all transcripts, codes, and emergent themes were reviewed by two authors for coherence and agreement (Whittemore et al. 2001). Any disagreements were resolved following in-depth discussion and re-examination of the data set. Peer debriefing was conducted to enhance the trustworthiness and credibility of the analysis (Morrow 2005). Peer debriefing took place with regular meetings held with co-authors for feedback regarding the interpretation of the data and possible instances of bias. Anonymised participant comments are provided to illustrate our findings and all participants have been assigned a pseudonym.

#### Questionnaire Measures of Child PTSD Symptoms

Parents completed the parent version of the UCLA Posttraumatic Stress Disorder Reaction Index (UCLA-RI; Pynoos et al. 1998) in relation to their child’s exposure to trauma and subsequent PTSS. The child and adolescent versions of the UCLA-RI (Pynoos et al. 1998) were administered to young people in the study in order to obtain their own reports of PTSD symptoms. The UCLA-RI is a commonly used measure of child and adolescent PTSS and has been found to correlate highly with a diagnosis of PTSD (McDermott and Cvitanovich 2000; Steinberg et al. 2013), and to show good internal consistency and test-retest reliability (Steinberg et al. 2013; Steinberg et al. 2004).

## Results

In our final sample parental age ranged from 34 to 55 years and 71% were mothers. Child age ranged from 8 to 15 years and 71% of participating children were male. Demographic and trauma characteristics are described in Tables 1 and 2.

**Table 1:** Participant demographic information

Demographic characteristic	Mean/Proportion
Child mean age	11.4 (2.3 SD)
Parent mean age	41.3 (7.8 SD)
Parent marital status	
Married	71.4%
Cohabiting	28.6%
Mean number of traumatic events experienced (Parent report)	1.7 (1.5 SD)
Mean total UCLA-RI Score (Parent report)	29.2 (15.8 SD)
Mean income	
£20,000–39,000	28.6%
£40,000–59,000	14.3%
£50,000–69,000	14.3%
£100,000–119,000	14.3%
> £200,000	14.3%
Ethnicity	
White British	85.7%
Asian British	14.3%

*UCLA-RI* UCLA Posttraumatic Stress Disorder Reaction Index, *PTSD* overall severity score reported, *SD* standard deviation

**Table 2:** Participant trauma characteristics

Parent pseudonym	Parent interviewed	Parent age	Child age	Child gender	Trauma experienced	UCLA-RI severity score (Parent report)	UCLA-RI severity score (Child report)
Nora	Mother	34	11	Male	Witnessed domestic violence	40	N/A
Aubrey	Mother	46	13	Female	Traumatic medical procedure	23	56
Amala	Mother	55	12	Male	Physical assault	47	N/A
Patrick	Father	38	15	Male	RTA	39	32
Patrick	Father	38	12	Male	RTA	N/A	8
Francis	Mother	36	11	Male	Witnessed domestic violence	22	10
Lois	Mother	39	6	Female	RTA	4	13

*UCLA-RI* UCLA Posttraumatic Stress Disorder Reaction Index, *PTSD* overall severity score reported, *N/A* data unavailable as parent did not complete or parent refused for child to take part, *RTA* road traffic accident

### Results of Thematic Analysis

As delineated in Table 3, four key themes emerged from the data, reflecting parental experiences and efforts to support their child following trauma exposure.

**Table 3:** Themes and sub-themes following thematic analysis

Theme and sub-themes
Post-trauma Perceptions of the Child
Understanding of children’s coping via behavioural cues
Perceptions of the child as having experienced significant negative changes
Child’s post-trauma distress an isolated change
Use of behavioural comparisons to determine child coping
Gradual improvements in child recovery
Scaffolding Discussions of the Trauma and Associated Distress
Encouraging openness
Parental Warm Support
Reassurance
Addressing the child’s trauma-related anxiety
Parental encouragement of a positive perspective of the trauma
Mixed messages of safety and simultaneous encouragement of vigilance
Efforts to resume children’s routines
Addressing the child’s negative appraisals
Encouraging Avoidance
Removal of child from contact with trauma reminders
Parental advocacy of avoidance as a coping strategy to prevent child distress
Perception of and Involvement in Treatment Sought for Post-Trauma Difficulties
Barriers to psychological treatment
Psychological treatment experienced as helpful in addressing child recovery
Psychological treatment experienced as unhelpful with no child recovery gains
High levels of parental engagement in treatment
Psychological treatment provides parents with insight into child experiences of trauma
Psychological treatment assists parents in caring for their child
Desire for further information about child recovery and coping
Need for additional support during “crisis points”
Impact of Trauma on the Parent
Parental post-trauma helplessness and anxiety
Parental blame of others or self-blame
Parental use of avoidance as a coping strategy
Parental reinstatement of pre-trauma routines as a coping strategy
Parental positive psychological changes post-trauma
Parental psychological treatment experienced as helpful
Parental experiences of social support

#### Post-Trauma Perceptions of the Child

Parents often described their children as having profoundly changed following the trauma and parents understanding of how their child was coping after the event was often informed by their behavioural cues. Most parents were deeply concerned by their child’s change in behaviour post trauma, and four parents viewed their child as having experienced profoundly negative changes in their personality or demeanor. However, in two cases where children experienced single, isolated manifestations of post-traumatic distress (e.g. vomiting, nightmares), children were considered by parents as essentially unchanged.

*She had problems with her behaviour…she went really quiet, which is not like her as she’s very chatty, she can talk so much, but she went really quiet. She didn’t want to be around people… She does mental checks like when we get into the car she will be like “right have we got this, that, the other” …and then if her brother is being really loud and naughty she will say to him “you don’t want us to have another crash happen do you? You need to let [mum] concentrate” … she shouldn’t really be like that, so I just really was concerned… and I’m like oh God she shouldn’t be thinking like that because she’s only a child. (Lois, mother, 39 years)*

Parents compared their child’s pre- and post-trauma behaviours or compared their post-trauma behaviours to the behaviour of other children to determine whether their child was functioning ‘normally.’ Parents additionally sought others’ (e.g. teachers) impressions of their child’s coping for external validation.

*They were both just normal happy kids before…I think Charlie has to work at being sort of a bit upbeat about things now, it seems like it’s a bit of an effort for him to be happy about things. That’s sort of how he comes across, his whole demeanor is sort of quite down…he doesn’t have as much enthusiasm to do things as he did before, he doesn’t have the same sort of confidence and drive that he did before. (Patrick, father, 38 years)*

In many cases, a considerable amount of time had passed since the trauma and parents reported that their children had since made gradual improvements in their behaviour and recovery.

*I think he's anyway much better than before because straight after that situation that happened and I can remember he was very, very angry…when it was bath time or something he’d kick the wall and he was very angry, very frustrated every single day…now he's more calm and he changed his behaviour definitely. We’ve still got some problems but it’s not like before, he's much better now, I think…it’s because the time and [also]I think because he feels secure in his family. (Nora, mother, 34 years)*

#### Strategies to Support the Child

Parents were very sensitive to their child’s post-trauma distress and reported supporting their child with strategies that were informed largely by intuition and instinct. Five parents were directly exposed to the traumatic event and experienced significant physical injuries and psychological distress as a result. Despite this, parents went to considerable lengths to support their child, putting their child’s needs before their own.

*You just have to put on a brave face and it’s just you’re not reassured yourself and you have to convince them, it’s just you’re being strong for them really…you do tell them these sort of things don’t happen often, you just tell them that [and] you have to convince yourself as well. (Lois, mother, 39 years)*

The following sub-themes were identified.

#### Scaffolding Discussions of the Trauma and Associated Distress

Parents encouraged their children to feel that they were available if they wanted to talk about the event or their feelings. Some parents reported that their child would bring up the traumatic event unexpectedly in conversation and this was often interpreted as a sign that their child was emotionally ready to discuss the trauma. Parents would respond by actively listening to and engaging with their child to facilitate a familial atmosphere of openness where trauma-related discussions were welcome.

*I think my initial reaction as a parent to something like this would have been to try keep the kids remote from it, you know, to protect them… but because both the kids were there, they both saw what happened… so we couldn’t shelter our kids from any of that, we couldn’t do that as parents. So from very early on we talked about it and we talked about it a lot. If the kids wanted to talk about it at all, didn’t matter what time it was or if we were having a conversation about something else, it wasn’t off limits. (Patrick, father, 38 years)*

Parents made several efforts to facilitate their child’s discussion of their posttrauma distress, including teaching their children words to better articulate their anxiety, encouraging their children to use analogies to facilitate discussion of their feelings, and holding trauma-related discussions in environments where their child felt safe. Parents also encouraged their children to feel that it was normal to experience distress following the trauma.

*He felt safe in the car and driving forwards…he always opened up, he always got something off his chest every single day in the car, it was one of the biggest therapies we did with him…we talked about all the things that had happened to him as far as he could say them and how he felt as far as he could articulate it. He did find that very, very hard to put names on feelings…so we tried to do things by analogy by saying you know “I sometimes felt blah blah blah, was it like that?” ...so we got out as much as we could. He talked a lot about fear and everything that made him frightened … we taught him the phrase ‘hyper-vigilant.’ (Amala, mother, 55 years)*

#### Parental Warm Support

Children often experienced considerable anxiety posttrauma and parents supported their children by offering reassurance, encouraging children to feel safe, and normalizing the trauma. To address children’s anxiety, parents also encouraged their child to face their fears through exposure to anxiety provoking situations and organized confidence building activities to foster their child’s self-esteem. Parents also promoted a positive perspective of the traumatic event, for example, by emphasizing that negative events may not recur in the future. .

*We just I think tried to make him feel first of all safe, whether that was physically or emotionally safe…we used to try and make him feel very safe in the house and very safe with us and we would try and make him calm by whatever means, even things like massage or lighting or we bought him a kitten, anything. (Amala, mother, 55 years)*

However, depending on the circumstances, some parents simultaneously emphasized that the family were not entirely out of danger and encouraged children to be vigilant and prepared for the worst case scenario. Such mixed messages appeared to stem in part from parents’ own anxiety and concerns post-trauma.

*He would come up to me and say “what happens if [the perpetrator is] not sentenced? What happens if he’s not found guilty?” Then I said “we will have to run, we will have to leave the school here and we will have to actually just up and go and leave everything here” … that’s the reality actually. I do realize that if he’s not sentenced erm then we’ll have to leave [home] and we will have to leave the schools again. (Francis, mother, 36 years)*

To facilitate their child’s recovery, parents also made concerted efforts to resume their child’s pre-trauma routines, which was thought to be helpful and reassuring for their child. As seen in this extract, parents often went to considerable lengths to bring a sense of normality to their child’s daily life, often at significant personal cost due to parents own physical restrictions and injuries. However, in several cases parents reported difficulty resuming pre-trauma routines due to their child’s ongoing posttraumatic distress and significant symptoms.

*We tried to stick to their routine… and make sure that I was there and then try and make myself go to the school, so I would get a taxi to the school and collect them…just to offer them reassurance I would collect them and then get the taxi back, so I started doing that so it’s been a gradual thing but they’ve been much better. (Lois, mother, 39 years)*

Parents of several children reported that their child held negative appraisals, such as self-blame, following the trauma. Parents often viewed these negative appraisals as unhelpful to their child’s coping and attempted to support their children by encouraging them to accept what had happened or reassuring them that they could not have prevented the event.

*[David] blamed himself for not checking the bag…he was telling me once “I should have checked the bag and I should have known that the knife was there” … [he thinks] he should have checked the bag and all that could be avoided. “No” I said “no, no, no, no…that’s not your fault, it was supposed to happen on that day and that’s it, that’s what happened” I said “no, no that that is definitely not your fault and just stop thinking about it.” (Francis, mother, 36 years)*

#### Encouraging Avoidance

Parental encouragement of trauma-related discussions was not universal and several parents promoted both cognitive and behavioural avoidance strategies to cope with the trauma. Parents removed their child from contact with trauma reminders as these were thought to be harmful and to contribute to their child’s distress.

*We make some [changes] because like he can’t watch…some programs on TV, you know, if it’s something on TV[or] he hears on the news like about bad accident like someone kill anyone or sometimes like the parent kill their children or something like that or about child abuse or something like that, we always switch off the program or just change the channel quickly because I don’t want him to see that because I think [it will] scare him, he can remember what happened or maybe he can think [about it] again. (Nora, mother, 34 years)*

In particular, one parent attempted to ‘over-ride’ or fade their child’s trauma-related memories by removing their child from the scene of the trauma and attempting to foster particularly positive memories in the weeks following the event.

*I think [David] was supposed to start [psychological treatment] in April…[but] I wanted to take them away on holiday to my family…I think we flew [overseas] on the fifth of May and that kind of delayed… the beginning of the sessions. But that point I thought…it will do them more good if they would spend time with my family there and with me and [my spouse] so we flew all of us [overseas where] they have only nice, happy memories…I took them out of this town and we went to the place where we have lots of happy memories…once they came back it was a bit faded the memories because [of] all the happy things and everything that happened [overseas]with them for these four, three weeks…it’s probably like override what ever happened on that day.(Francis, mother, 36 years)*

A number of parents reported avoiding discussion of the event to prevent their child becoming distressed or reminding them of the traumatic event. Parents avoided talking about the trauma until their child initiated the conversation and encouraged other family members to adopt the same approach. Such avoidance of discussion reflected parents’ own concerns that they did not know whether discussing the trauma with their child would improve or worsen their child’s post-trauma difficulties; avoidance of discussion seemed the safer choice faced with this uncertainty.

*[My sister] never spoke with Lewis again because she was asking him about [it]and I said to her “if Lewis doesn’t mention then I think it’s maybe better [if you] don’t speak…if he wants to say something you can talk with him, but you don’t have to start this subject” because it’s difficult, I don’t know what is better for him[to] just talk about what happened, or just don’t talk and then it will be forgot about… maybe he never forgot about it, yeah maybe if I start to talk about what happen maybe he [will] think [about it] much more, maybe this will be worse for [him]… I’m not sure. (Nora, mother, 34 years)*

#### Perception of and Involvement in Treatment Sought for Post-Trauma Difficulties

Psychological treatment was described as difficult to access in many cases and parents often reported that their persistence was instrumental to their child receiving treatment for their post-trauma difficulties. Barriers to accessing psychological services included: parents being unaware of available services; not being automatically referred after voicing concerns to the GP about their child’s adjustment difficulties; extensive waiting times for assessments; or infrequent therapy sessions. As seen in the following extract, it was thought that a physician responded to their child’s physical injury but they did not appreciate the emotional consequences of the traumatic event. Parents’ experience of difficulties in accessing treatment for their child in one case resulted in parental concerns that they may be over-reacting in response to their child’s post-trauma difficulties, contributing to parental anxiety and feelings of uncertainty about how to best support their child.

*I went to the doctors about their behaviour…several times … and then I went to another GP and then he automatically referred me … and I was like why couldn’t you have given me this earlier? It has taken them…eight months...I’m not sure whether because there wasn’t anything physically wrong with them that this didn’t get noticed, because it was all emotional and I don’t think they see the emotional part … I think if it was something physical the doctors would’ve said “oh, OK, yeah, yeah, yeah.” (Francis, mother, 36 years)*

Once psychological treatment was accessed, many parents perceived the treatment received as helpful in addressing their child’s adjustment difficulties and providing an opportunity for their child to discuss the trauma or receive coping advice. In a few cases, parents reported that the treatment their child had received was unhelpful, and did not address what parents perceived to be the root cause of their child’s adjustment difficulties or lead to an improvement in their symptoms. In such cases, different treatment plans were discussed with therapists and an alternative treatment approach was adopted to better meet their child’s needs.

*The first therapist tried CBT, but he was beyond CBT. The questions were too invasive for him and made him worse. So he needed very, very gentle therapy from someone who would just connect with him and get his trust and during that fortnight…he had I think five or six session…which just somehow got him grounded. (Amala, mother, 55 years)*

Parents were often very involved and engaged in the treatment itself, participating in children’s treatment activities and attending family therapy sessions, which provided insight into their child’s feelings and distress following the trauma. In one case where several family members were involved in the traumatic event, family therapy was considered particularly helpful as this provided the opportunity for parents and children to share their post-trauma distress. This fostered a sense of familial support and acceptance that was thought to be instrumental for recovery. Child treatment that facilitated trauma-related discussions also provided parents with further details regarding the traumatic experience and its sequelae which deepened parents’ understanding of their child’s traumatic experience.

*I didn’t realize until [the clinician] came along… I thought I actually knew how she was feeling but I didn’t really which is quite sad for me as a parent, not knowing how she actually really felt, because I thought she told me a lot of things but to be honest she’d bottled quite a lot of her feelings up. There were certain things that she’d noticed that I hadn’t even noticed as well during the [accident] so it was only…after [she] got referred…and they did [a] sequencing activity, it was only then that she actually said well [the driver] had a necklace on with a [star] on which I didn’t even know, so there’s things that [she] saw that I didn’t see. (Francis, mother, 36 years)*

Parents felt considerable anxiety about how to best support their child’s recovery and receiving confirmation from expert therapists that the strategies they were using were effective was a source of reassurance. Where therapists provided guidance to parents on activities to do with their children in-between therapy sessions, this helped parents to feel actively involved in their child’s psychological recovery and reduced feelings of helplessness and anxiety about how to best care for their child. Taken together, children’s psychological treatment was often considered a valuable source of support and guidance for parents, helping them to provide and be confident in delivering support to their child post-trauma.

*[The clinician] came out to see us and that… was extremely valuable because again as a parent [you’re] trying to just fudge through it really as best you can and it was helpful [to] me personally to have someone come out that was experienced at this sort of thing and actually say “do you know what? You’re doing alright, you’re doing everything that you can feasibly do and you’re coping with it as best you can.” (Patrick, father, 38 years)*

Conversely, in some cases parents held expectations that they would be given advice and strategies about how they could best support their child post-trauma and expressed disappointment in the process from their perspective when such guidance was not received. Similarly, parents experienced frustration when their child’s school teachers or Special Education Needs Coordinator (SENCO) were reportedly aware of their child’s psychological adjustment difficulties but did not volunteer advice or a referral to facilitate access to formal psychological services.

*It wasn’t so helpful for me because, you know, I think will be more helpful if [the clinician gave me] some clue of what can I do or…how can I talk with him…she didn’t say anything like that and I was a little disappointed…she just send a letter with some website pages or books that I can read about the children…but not any help…because if you read the books it’s just very …abstract information, you can’t say oh it’s good for your child because every child is different and have different experience, and I think… she was sat with Lewis and with me and she knows the situation she can help much better because she knows the child, but books it’s not the same. (Nora, mother, 34 years)*

Overall, parents described a desire for information about what child behaviours or responses to look out for as signs of poor post-trauma coping and advice to help them better understand their child’s experience. Some parents reported acute “crisis points” where their child became severely symptomatic for several weeks and parents felt unable to provide the support that their child urgently needed. As urgent support from psychological services was reportedly inaccessible, parents viewed Emergency Departments (EDs) to be their only available option at these times. During periods of acute symptoms, parents reported needing further information about how to best support child coping and additional, more flexible support from professionals.

*The most the single most useful thing…is somebody on the phone… to talk to daily if needs be to say you know… “We can’t calm him down, what do we do? Is this normal for someone who’s got PTSD?” …so, I would say a helpline for us because we needed to help him and it was it was 24-hour care. (Amala, mother, 55 years)*

#### Impact of the Trauma on the Parent

All parents reported experiencing significant distress post-trauma. Parents reported feelings of blame towards themselves or others for causing the event. Parents also blamed themselves for being unaware of their child’s symptoms or for their perceived contribution to their adjustment difficulties. For example, a parent who reacted to their child’s trauma with considerable fear and horror blamed themselves for potentially contributing to their child’s distress at the time of the event and their subsequent development of PTSD.

*I think if [her father] had been there [instead] when she was unwell…he is much better at dealing with blood and when people are ill he doesn’t panic, it frightens me, it doesn’t frighten him… if [she does have] PTSD then I think that that me screaming out for help…almost certainly will have not been helpful and that [he] would probably have just…said “don’t worry, somebody’s coming.” … I think he just would have been calmer and then she would have been calmer. (Aubrey, mother, 46 years)*

To cope with their own feelings, several parents used avoidance based strategies. Parents avoided trauma reminders and locations associated with positive pre-trauma memories as such places evoked distress due to appraisals of permanent familial change. Additionally, parents avoided discussing the event outside of the family due to concerns that others would blame them for the trauma.

*We’ve got some friends here but actually … they don’t know my life and Lewis’ life actually because we never talk about it with other people…because I don’t like to speak with someone who knows me because I think, you know, they treat me or they look at me [differently]…maybe they say I’m not good mum…for me it’s difficult to speak about this situation because sometimes I think that people can say “oh it’s your fault.” (Nora, mother, 34 years)*

In order to cope with the trauma, parents often had to come to terms with and accept their own physical injuries and limitations. Resuming their own pre-trauma routine and activities was thought to be helpful in coping with the event. Parents also experienced positive psychological changes following the trauma, including greater awareness of and sympathy for others’ distress; greater appreciation of their child; and gratitude that their and their child’s traumatic event and/or injuries were not worse.

*Before something like this happens to you, you know, you see something on the news and for about two or three minutes you think “oh that’s terrible, absolutely terrible” and then you carry on. But when something like that does actually happen to you…you’ve got a complete empathy for other people that it’s happened to because you know how they feel, you know actually what it means for them to go through it. (Patrick, father, 38 years)*

Several parents reported that counselling was instrumental to their recovery as it provided an opportunity to voice their concerns in private, away from their children, to someone who would not judge them.

*I had counselling from work and then I had CBT… it helped to talk to an outsider that didn’t know me and wasn’t going to judge me… so she just helped me accept things and she listened to me and then I’d tell her how I was worried about I’m not gonna have a job left and, you know, I didn’t want the kids to know. (Lois, mother, 39 years)*

However, whilst parents were in contact with several agencies, including GPs, CAMHS, and social workers, to arrange counselling for their child, some parents were not referred to psychological services themselves despite their own significant post-trauma distress.

*I would have definitely loved some sessions and [psychological treatment] for [my spouse] something professional, on a professional level, because he is the one who is actually affected more than probably me. I could have used probably the help, some sort of [help] just to talk through these things… I didn’t have anything…If somebody would give us a hint, not a hint, [but] kind of like an address or phone number of an organization who could help us actually in a professional way then I’d say that would be helpful. (Francis, mother, 36 years)*

Parents reported receiving social support from their spouse, extended family, and friends and most felt that such support was readily available if needed. Social support included practical care to help parents cope with their physical injuries post-trauma, frequent visits or messages from friends, prayers, and reassurance that their child would recover.

*My mum has always been there all the time, she’s been there from day one… and my mum’s been praying for a good outcome and things like that…[and] I’ve had friends that have supported us… they’ve offered to cook, not that I’ve needed it, but it’s just being there. Someone just picks up the phone and asks you how you are, you know, bringing the crossword round to keep you entertained… so you know who your friends are and the people that are there when you need them. (Lois, mother, 39 years)*

At the same time, social support could also be somewhat unhelpful. Some parents reported that their family had received a great deal of social support immediately post-trauma; however, this support lasted only a few weeks at which point families felt they were expected to have moved on from the event.

*I think the difficulty is that when these things happen you tend to get a massive influx of people within the first probably month, well probably less than that, the first two or three weeks. Everybody is really sympathetic, they want help, they want to tell you how sorry they are and but that does drop away really, really quickly and people kind of then move on with their lives and they expect you to do the same and if you’re in a situation where you can’t actually move on then that’s difficult. (Patrick, father, 38 years)*

## Discussion

The aim of the present study was to investigate the experiences of parents in caring for their child following trauma exposure and explore parental perspectives of their child’s post-trauma psychological treatment in order to inform clinical practice. The narratives uncovered a key theme relating to strategies used by parents to support their children. Parents attempted to support their children using three core strategies: promoting avoidant coping, scaffolding trauma-related discussions, and providing warmth. However, underlying these strategies of support were feelings of considerable parental anxiety and helplessness to adequately and appropriately care for their child’s significant post-trauma distress. Contributing to parents’ distress were the considerable barriers faced in accessing psychological treatment for their child, as well as perceptions that the provision of guidance and support from therapists about how to best support their child were often inadequate. Given the challenges faced in accessing and engaging with psychological treatment, it is important to critically evaluate the support strategies used by parents to foster child adjustment.

Parents’ anxiety contributed towards the use and advocacy of avoidant coping strategies in several cases. Their uncertainty as to whether discussion of the trauma may worsen their child’s post-trauma difficulties meant discussions of the event with the child were often limited. Following a traumatic event, such reactions are common and understandable; however current theories also emphasize the role of avoidance in the development and maintenance of PTSD (Ehlers and Clark 2000). Previous research has found parental attitudes favoring avoidant coping to be associated with child PTSD severity at six-months post-trauma (Ehlers et al. 2003) and children’s use of maladaptive cognitive strategies, including thought suppression, are a leading risk factor for PTSD (Trickey et al. 2012). Whilst the efficacy of particular coping strategies may depend on a variety of factors, including time since the trauma, appraisals of the event, and other available resources (Compas et al. 2001; Dempsey 2002; Joseph et al. 1997), parental encouragement of avoidant-based strategies to children engaged in psychological treatment post-trauma represents an important consideration and target for future investigations.

Parents also made efforts to support their children in a number of warm, positive ways, including offering reassurance and going to considerable lengths to make their children feel safe. This use of warm support is consistent with the support strategies reportedly used by parents of children who have attended EDs following trauma exposure (Williamson et al. 2016). As high levels of parental support are associated with fewer child PTSD symptoms (e.g. Bokszczanin 2008), such supportive parental responses may contribute positively to the child’s adjustment.

Several parents also encouraged their child to discuss the traumatic event and attempted to facilitate their child’s disclosure of their post-trauma distress. Trauma-related discussions between parents and their child have been found to be associated with fewer child PTSD symptoms as they may lead to an improvement in the coherency and completeness of the child’s trauma memory (Fivush et al. 2003; Salmon and Bryant 2002). This may positively influence child adjustment, as an incomplete and poorly elaborated trauma memory is thought to be associated with the development and maintenance of PTSD (Ehlers and Clark 2000). Parents also sought to address children’s potentially maladaptive appraisals of blame and promoted a positive perspective of the event. As maladaptive cognitions contribute to the continuation of child PTSD symptoms (Meiser-Stedman et al. 2009; Meiser-Stedman et al. 2007), such positive reframing coping advice may support functional reappraisals in children by rectifying misinterpretations of the trauma, thus leading to more adaptive coping following a traumatic event (Kassam-Adams and Fein 2003; Kilmer and Gil-Rivas 2010; Salmon and Bryant 2002).

Parents often had to overcome a number of barriers in order to access psychological treatment for their child and felt that their persistent involvement was instrumental to attaining treatment. The reported difficulty in accessing formal psychological services is consistent with the limited literature on the subject (Coyne et al. 2015). It is notable that a number of parents did not receive a referral to psychological treatment to address their own post-trauma distress, despite contact with several health professionals to arrange treatment for their child. The presence of parental PTSD predicts child PTSD (Morris et al. 2012; Spell et al. 2008) and, although previous parental trauma exposure was not assessed in this study, it is possible that parents’ own prior experiences of trauma may have influenced their responses to their child following child trauma. For example, Moehler et al. (2007) found that parents with a history of abuse engaged in more intrusive parenting behaviours than those without a history of abuse. This may have clinical implications not only for child adjustment but also for the engagement of families in psychological services or interventions post-trauma.

Several expectations about psychological services were held, including the parental belief that they would receive guidance about how to best support their child. However, parents reported feeling unprepared about how to manage their child’s symptoms, particularly when symptoms became acute, and additional support and information from psychological services was desired. This suggests a need for the delivery of additional guidance and flexible support for families from mental health professionals where children are experiencing particularly acute crisis episodes following trauma exposure.

As parents experienced significant anxiety about how to best care for their children and support their psychological recovery, receiving confirmation from a therapist that their supportive strategies would promote child adjustment was also particularly useful. Recent research has extolled the importance of parental engagement in child psychological treatment post-trauma (Cobham et al. 2016) and parent and child satisfaction with services is strongly associated with treatment completion and functional improvement (Garland et al. 2007; Oruche et al. 2014; Ronzoni and Dogra 2012). Therefore, clinical care and psychological interventions may be enhanced by the inclusion of targeted information and advice for families engaged with psychological services following child trauma exposure.

This study had several strengths and weaknesses. Among the strengths was the inclusion of parents of children who experienced a range of trauma types. Furthermore, parents’ views were collected after varying lengths of time post-trauma which allowed for the examination of parental experiences from diverse circumstances. The relatively small number of cases also allowed for in-depth analysis and thematic saturation was achieved (Crouch and McKenzie 2006; Marshall 1996). Among the weaknesses is the limited diversity of the sample and the recruitment of mostly mothers. Future studies could include the perspectives of more male caregivers. Finally, all children recruited to this study accessed psychological treatment and the views of parents whose children were not successful in accessing treatment for their post-trauma difficulties were not included.

Despite these limitations, the present study provides some of the first evidence of the strategies used by parents of children who accessed psychological treatment to support child adjustment. The results expand on previous research examining parental experiences and strategies used to support children following EDs attendance after single-incident trauma exposure (Williamson et al. 2016) and provide insight into the parental perspectives of caring for a child with clinically significant post-trauma distress. Future research is needed to examine the psychological impact of such support strategies and the implications for child adjustment in families where the child is receiving formal psychological support. These findings also illustrate parents’ expectations and experiences of their child’s psychological treatment, including the significant difficulties faced in accessing psychological services following trauma. This suggests a need for not only more accessible psychological treatment but also the provision of targeted information and advice to parents, which may help to address their concerns and improve the overall family experience following trauma exposure.
